# Jensen’s Inequality and the Impact of Short-Term Environmental Variability on Long-Term Population Growth Rates

**DOI:** 10.1371/journal.pone.0136072

**Published:** 2015-09-09

**Authors:** Evan J. Pickett, David L. Thomson, Teng A. Li, Shuang Xing

**Affiliations:** 1 School of Biological Sciences, University of Hong Kong, Hong Kong, China; 2 Biology Department, College of Science, UAE University, Al Ain, United Arab Emirates; Tennessee State University, UNITED STATES

## Abstract

It is well established in theory that short-term environmental fluctuations could affect the long-term growth rates of wildlife populations, but this theory has rarely been tested and there remains little empirical evidence that the effect is actually important in practice. Here we develop models to quantify the effects of daily, seasonal, and yearly temperature fluctuations on the average population growth rates, and we apply them to long-term data on the endangered Black-faced Spoonbill (Platalea minor); an endothermic species whose population growth rates follow a concave relationship with temperature. We demonstrate for the first time that the current levels of temperature variability, particularly seasonal variability, are already large enough to substantially reduce long-term population growth rates. As the climate changes, our results highlight the importance of considering the ecological effects of climate variability and not just average conditions.

## Introduction

Population growth rate is fundamental to understanding the relationship between populations and environmental conditions [1]. Population growth is determined not just by the well-documented effects of average environmental conditions [2–4], but also by more complex effects of environmental variability [5]. This may include the well-known effects of variability on extinction risk [6, 7], with fluctuations reducing populations to the critically low numbers where they become vulnerable, but in theory environmental variability can also impact the long-term growth rates of wildlife populations more directly [8, 9]. This theory is grounded in the classical Jensen’s inequality [10], where for nonlinear functions, *f*(*x*), the average of the function, $\bar{f(x)}$, is not equivalent to the function of the average, $f\left(\bar{x}\right)$. From the theory, we know that the direction and magnitude of this inequality can differ markedly depending on the nature of the function and the degree of variability. The average of concave-down functions is lower, but the average of convex functions is higher and the average of functions that switch concavity could be either, so the magnitude of the inequality is determined not just by the variability but also by the degree of concavity in the function [10, 11].

When fluctuating conditions drive variability in population multiplication rate ‘λ’, Jensen’s inequality can introduce an inherent negative pressure on long-term population growth ‘r’ because the relationship between ‘r’ and ‘λ’ is concave down: r = ln(λ) [9, 12]. For example, a population that declines by 50% in one year does not fully recover if it then increases by 50% the next. Although the arithmetic mean of the population multiplication rate in this instance is (0.5+1.5)/2 = 1, the geometric mean is only (0.5*1.5)^1/2^ = 0.866 and the average population growth rate is negative because the mean of the function (r = lnλ) is below zero. In the long term, this variability produces populations which are smaller than they would otherwise be, and this generalises for any population that experiences variability in its rate of multiplication.

However, the impact of variability is not only mediated through this relationship between r and λ; Jensen’s inequality is relevant to any nonlinear relationship between environmental variables and population growth rate [11, 13]. The mathematical proof for this is over a century old [10], and although theoretical biologists have long recognised its relevance for populations in fluctuating environments [8, 9, 14, 15], there have been few attempts to test the theory’s importance by quantifying the amount by which environmental variability actually reduces wildlife population growth rates in practice.

A study of phytoplankton showed that when population growth rates increase exponentially with temperature, this convex relationship can actually result in a small positive effect of temperature variability on the population growth rate [13]. Convex relationships can occur, most notably in ectotherms living well below their optimal temperatures (Amarasekare & Savage 2012), but more commonly, species respond with concave functions [4, 16]. When functions are concave, it is known from theory that variability will have negative effects, but it is not yet clear whether these negative effects will actually be large in practice. A small number of studies have quantified the effect of inter-annual variability on population growth rates and found them to be small [17, 18], but much of the variability in environmental conditions is intra-annual rather than inter-annual and it is not yet known whether this shorter-term variability has any appreciable effect on rates of population growth.

Here, by developing models which allow us to partition the effects of annual, seasonal, and daily temperature variation, we aim to test whether these environmental fluctuations can reduce wildlife population growth rates in practice.

We apply these models to long-term data on the Black-faced Spoonbill (*Platalea minor*), an endothermic study species in which population growth rate shows a characteristically symmetrical concave-down relationship with temperature, and while we find that inter-annual variability in temperature may indeed have only a small effect on population growth rates, we show that the seasonal and daily variability can have a major impact, reducing population multiplication rates to around half of what could be achieved under constant conditions.

## Materials and Methods

This research is based on pre-existing non-human data which are freely available in the public domain (http://www.hkbws.org.hk/).

In order to quantify the amount by which prevailing levels of environmental variability can reduce wildlife population growth rates, we first needed to select a suitable study system, and reconstruct the relationship between population growth rate and some measurable component of the environment. We chose to focus on the relationship between temperature and the population growth rates of Black-faced Spoonbills (*Platalea minor*). *Platalea minor* is an endangered species whose population has been well monitored, with teams in Hong Kong conducting systematic population counts every year since the species established itself there in 1985 as a regular winter visitor to the wetlands of Deep Bay [19]. Since then, the Hong Kong population has increased from 11 to 188 birds, with fluctuations ranging between 7 and 479 (see S1–S3 Files for full data set). Over this period, we were able to reconstruct the relationship between population growth rate and temperature by combining these long-term population data with long-term temperature data from the NCEP/NCAR reanalysis [20]. *Platalea minor* is a migrant species, and while our study population has been surveyed on its wintering grounds in Hong Kong, it spends the summer breeding in western Korea [21]. Throughout the Hong Kong winter and the Korean summer, temperature data from the NCEP/NCAR reanalysis provide us with detailed information on the daily temperatures to which *P*. *minor* have been exposed, allowing us to integrate the overall effects of temperature on population growth rate throughout the year on both the wintering grounds and the summer breeding grounds.

The exact mechanism by which temperature affects population growth rate here is unknown, but it is likely to include numerous different components which may act directly or indirectly on the birds, and which may have either immediate or delayed effects. With our analysis we sought not to catalogue or tease apart these individual components but rather to model their combined action as they work together in one overall composite effect. Population growth rates are determined by the balance between birth rates and death rates, so any effect of temperature on either birth or death will affect population growth. The action of temperature is not limited to direct immediate effects of temperature on reproductive performance during the breeding season, so we sought to build models which would also include any effects of temperature on death rates at any time of year, and any delayed or indirect effects which temperatures throughout the year might have on reproductive performance. Direct and immediate effects of temperature are well-known, with increased mortality from both heat [22] and from cold [23], but there is also evidence that breeding performance during the summer months can, for example, be affected by feeding conditions during winter [24]. *Platalea minor* is a waterbird, which uses its long flat beak to catch a range of fish and invertebrates in the shallow waters of coastal mudflats. One study in Taiwan [25] found that Mullet made up about half their diet, but they are quite generalist feeders, and if adverse winter temperatures were to affect the availability of Mullet or other food then this could affect the body condition of *P*. *minor* and their subsequent ability to breed and survive. We built models which could accommodate not just any direct immediate effects of temperature on reproduction, but the overall cumulative action of any effects which temperature at any time of year might ultimately have on reproduction and on mortality rates throughout the year.

We chose to focus specifically on the effects of temperature and to quantify the impact of temperature variability on overall rates of population growth. Temperature is only one component of environmental variability, and if temperature variability alone has an appreciable impact on population growth rates, then the overall effects of all environmental variability combined will be larger still. Unlike many other types of environmental variation, temperature has been widely and accurately recorded throughout recent history, it shows high levels of short-term variability, its impacts have been relatively well studied, and predictions of temperature change are well modelled. There are many other aspects of the environment which could affect population growth but we have focused here on temperature because it provides us with a good model system with which to quantify the effects of environmental variability.

The effect of environmental variability rests on the existence of non-linearity in the underlying relationship, and having selected our study system, we were then able to reconstruct this relationship between population growth rates and temperature, and to test explicitly whether it was nonlinear. By then constructing various temperature scenarios which included and excluded the daily, seasonal, and annual components of variability in temperature, we were then able to use our nonlinear model to predict and compare average population growth rates in the presence and absence of temperature variability, thus quantifying the amount by which population growth rate could be reduced by the prevailing levels of variability in temperature.

Reconstructing the relationship between the population growth rates of *P*. *minor* and temperature involved a number of analytical steps. First, because any effect of temperature variability on population growth rates hinges critically on whether the relationship is nonlinear, we used a quadratic function:

$$r=\alpha +\beta T+\gamma T^{2}$$ (1)

to capture any nonlinearity and to formally test whether this nonlinear model was significantly better than a simple linear model. In this function, *r* denotes the per capita growth rate, *T* denotes temperature, and the parameters of the quadratic function are denoted as *α*, *β* and *γ*. This quadratic function allows population growth rates to increase with temperature when it is too cold, and to decrease with temperature when it is too hot.

In order to estimate the parameters α, β, and γ, we rearranged the expression as:

$$N_{t+1}=N_{t}e^{\alpha +\beta T+\gamma T^{2}}$$ (2)

i.e:

$$N_{t+1}=N_{t}e^{r}$$ (3)

where *N*_*t*_ is the population size in year *t*, and *N*_*t*+1_ is the population size in the following year. Here, *e* ^*r*^ is the population multiplication rate, *λ*, and therefore the population size in year *t+1* is simply the population size in year *t* multiplied by the population multiplication rate. By using this format, we could estimate the same parameters *α*, *β*, and *γ*, but we were able to work with population size as our dependent variable, and that could then be modelled using a standard over-dispersed Poisson distribution. Furthermore, by specifying a log-link function, and by specifying *ln(N*_*t*_ *)* as an ‘offset’, we were able to implement this model using the standard quasi-generalised linear modelling framework:

$$\text{ln}(N_{t+1})=ln\left(N_{t}\right)+\alpha +\beta T+\gamma T^{2}$$ (4)

which we fitted using the statistical software package R v2.15.3 [26].

To partition out the effects of annual, seasonal and daily temperature variability on population growth rates, we worked with daily temperature data and constructed models which could accommodate effects at each of these time scales. The annual population growth rate (r) is the addition of the growth rates at shorter time intervals throughout the year, and a model of annual population growth rate can be built up on that basis. Just as an annual survival probability can be seen as the product of survival in four consecutive seasons, so the annual population growth rate, *r*, can be thought of as the sum of the daily population growth rates throughout the year:

$$r_{annual}=\Sigma r_{daily}$$ (5)

Each of these daily population growth rates can be modelled as a quadratic function of temperature on each particular day, so

$$r_{annual}=\Sigma \left(\alpha_{daily}+\beta T_{daily}+\gamma T_{daily}^{2}\right)$$ (6)

If each day is allowed to have its own intercept (α), then population growth rate is also allowed to change daily for reasons which may have nothing to do with temperature, e.g a change in predation rate, food availability, or the onset of breeding. This concept of daily intercepts maintains the shape of the quadratic relationship through time, but acknowledges that the function will shift up and down on the y-axis when other effects impact population growth. In practice, when we fitted the model of annual per capita growth rate, we only needed to estimate the sum of these α values, and not all the separate values for each individual day. Indeed, because the annual per capita population growth rate for the whole year is the sum of the daily per capita growth rates, it simplifies to:

$$r_{annual}=\sum \;\alpha_{daily}+\beta \;\sum \;T_{daily}+\gamma \;\sum \;T_{daily}^{2}$$ (7)

thus by creating one variable which is the sum of the daily temperatures, and a second variable which is the sum of the squares of the daily temperatures, we condensed everything into a model with just two variables (ΣT and ΣT^2^) and three parameters (Σα, β, and γ). In this way, by building up a model which acknowledges that annual population growth rate is determined by the sum of daily population growth rates, we can look at how daily temperatures affect annual population growth rates, and quantify the impact of short-term temperature variability. The model allows us to see not only how annual population growth rate will be affected by a hot or a cold year, but also how it will be affected by a year when temperatures are variable or constant. The ΣT increases with mean temperature, but the ΣT^2^ will also increase with variability in temperature. Together, these components of the model can tease apart the impacts of mean temperature and temperature variability.

Note too, that by building the model in terms of how annual population growth rate is affected by daily temperature variability, we can accommodate delayed effects. An adverse temperature on a particular day need not necessarily have its adverse effect immediately, and by modelling annual population growth in terms of the cumulative effect of daily conditions, the birds can encounter and incur the cost of adverse conditions on one particular day but they need not necessarily book those costs until sometime later. If for example adverse winter temperatures reduce the availability of prey, and if that nutritional stress then dampens breeding success later that year, these models will be able to include those effects; acknowledging that winter temperatures can affect breeding performance but acknowledging too that the effect could be somewhat delayed.

In implementing the models, these temperature variables were constructed by combining daily mean temperatures for each day through the Hong Kong winter season, and through the Korean breeding season. To interpret what the parameters mean for temperature changes across the whole year and not just for one day, this model was also rescaled by using the mean temperature instead of the sum of temperatures, and by using the mean square of temperature instead of the sum of temperature squares.

In reconstructing the relationship between population growth rate and temperature, we controlled and corrected for any effect of temperature on the proportion of the population which is actually counted, *p*. In year ‘t’, the observed population size, *O*_*t*_, will be influenced not just by the true population size, N_t_, but also by this ‘detection probability’, *p*_*t*_:

$$O_{t}=N_{t}\times p_{t}$$ (8)

Temperature could affect detection probability, *p*_*t*_, if it influences either the visibility of the birds or their chance of being on the Hong Kong wintering grounds during the systematic programme of standardised mid-January counts. If we did not control for such an effect then it could bias the apparent relationship between temperature and population growth rate, but we dealt with this using models which acknowledge that the observed population multiplication rate is influenced not just by the ratio of the true population sizes but also by the ratio of the detection probabilities:

$$\frac{O_{t+1}}{O_{t}}=\frac{N_{t+1}\times p_{t+1}}{N_{t}\times p_{t}}=\lambda \times \frac{p_{t+1}}{p_{t}}$$ (9)

If detection probability were constant, then no matter whether the whole population was being detected or only a small fraction of it, the ratio of the detection probabilities would equal one and this term could cancel out. However, if temperature were to have some effect on whether these migratory birds come to Hong Kong and get counted, then this ratio of detection probabilities needs to be addressed. This was achieved by modelling detection probability as a function of temperature,

$$p_{t}=e^{a+bT}=K_{o}e^{bT}$$ (10)

and we inserted this into our model:

$$\frac{O_{t+1}}{O_{t}}=\lambda \times \frac{p_{t+1}}{p_{t}}=e^{\alpha +\beta \bar{T}+\gamma \bar{T^{2}}}\times \frac{K_{o}e^{bT_{t+1}}}{K_{o}e^{bT_{t}}}=e^{\alpha +\beta \bar{T}+\gamma \bar{T^{2}}+b\Delta T}$$ (11)

Using the year to year difference in mean seasonal temperature, we can thus control and correct for any effect of changing Hong Kong winter temperatures ($\Delta {\bar{T}}_{W})$ and changing Korean summer temperatures ($\Delta {\bar{T}}_{S})$ on the ratio of detection probabilities. Thus even if temperature does affect detection probability, we can still reconstruct the quadratic relationship between population growth rates and temperature, estimating the parameters α, β, and γ from data on observed population size rather than true population size:

$$O_{t+1}=O_{t}e^{\alpha +\beta \bar{T}+\gamma \bar{T^{2}}+b\Delta {\bar{T}}_{S}+c\Delta {\bar{T}}_{W}}$$ (12)

Note that the underlying quadratic relationship between population growth rate and temperature is not fitted as a direct relationship between observations of annual population growth and observations of annual temperature–the data are in the form of observed population sizes *O* in years *t* and *t+1*, mean daily temperatures, mean daily square of temperatures, and as the change in summer temperature between consecutive years and the change in winter temperature.

By estimating these quadratic parameters, α, β, and γ, we reconstructed the underlying relationship between population growth rate and temperature, and we were then able to quantify the impact of temperature variability on long-term population growth rates. We constructed different temperature scenarios for the period 1985–2013 and compared the ratio of the expected long-term geometric mean of population multiplication rates in the presence and absence of temperature variability:

$$\frac{\lambda_{1}}{\lambda_{2}}=\frac{e^{\frac{1}{n}\Sigma (\alpha +\beta T_{1}+\gamma T_{1}^{2})}}{e^{\frac{1}{n}\Sigma (\alpha +\beta T_{2}+\gamma T_{2}^{2})}}$$ (13)

Here λ_1_ and λ_2_ are the expected geometric mean of annual population multiplication rates throughout the *n* years of the study, when exposed to the temperatures *T*_*1*_ and *T*_*2*_ of Scenario 1 (variable temperature) and Scenario 2 (constant temperature), respectively. Scenario 1 was the observed temperatures during 1985–2013 in Hong Kong winters and Korean summers, while for Scenario 2, each day during this period was set to the mean temperature of Hong Kong winter or Korean summer for the wintering and breeding seasons respectively. In effect, we compared the mean of the function versus the function of the mean; using this ratio as a way of quantifying the magnitude of Jensen’s inequality. Note that variability in temperature will not actually affect the terms in either α or β, so provided the mean temperature is the same for the different temperature scenarios, the expression simplifies to:

$$\frac{\lambda_{1}}{\lambda_{2}}=e^{\frac{\gamma }{n}\left(\Sigma (T_{1}^{2}-T_{2}^{2}\right))}$$ (14)

and because the variance and standard error of γ have already been estimated, the delta method could then be used to estimate the variance and standard error of this ratio [27].

$$Var\;\left[\frac{\lambda_{1}}{\lambda_{2}}\right]=Var\left[\gamma \right]\times {\left(\frac{\lambda_{1}}{n\times \lambda_{2}}\times \Sigma \left(T_{1}^{2}-T_{2}^{2}\right)\right)}^{2}$$ (15)

As well as quantifying the overall impact of all temperature variability combined, we also used a similar approach to tease apart the different effects of daily, seasonal and annual temperature variabilities on population growth rates. Rather than predicting average population growth rate using a scenario based on observed daily temperatures, we constructed scenarios which contained only daily, seasonal, or yearly variability in temperature, and we then compared the population growth rate predicted under these scenarios with that predicted under Scenario 2 with constant average temperatures.

Annual variability in temperature was built into a scenario where the daily winter temperatures from 1985–2013 were set to the mean temperature of each winter in Hong Kong, and the daily summer temperatures were likewise set to the mean temperature of each summer in Korea.

For seasonal variation we used the linear model procedure of R v2.15.3 [26] to construct quadratic models of Hong Kong daily winter temperatures against date through the Hong Kong wintering period, and Korean daily summer temperatures against date through the Korean breeding season. We then built the scenario using the expected value of temperature for each winter day and each summer day.

For the scenario involving daily variability, we first constructed a model with similar quadratic relationships of temperature against date and with a further additive effect of ‘year’ as a factor. We then took the residuals from this model and added them to the overall mean temperatures for the Hong Kong winter period and for the Korean summer period.

The population growth rates predicted under each of these specific scenarios were then compared to those predicted under the constant average temperatures of Scenario 2, and any difference was quantified using the ratio of geometric means as above (Eq 13 and 14).

## Results

**Fig 1:**
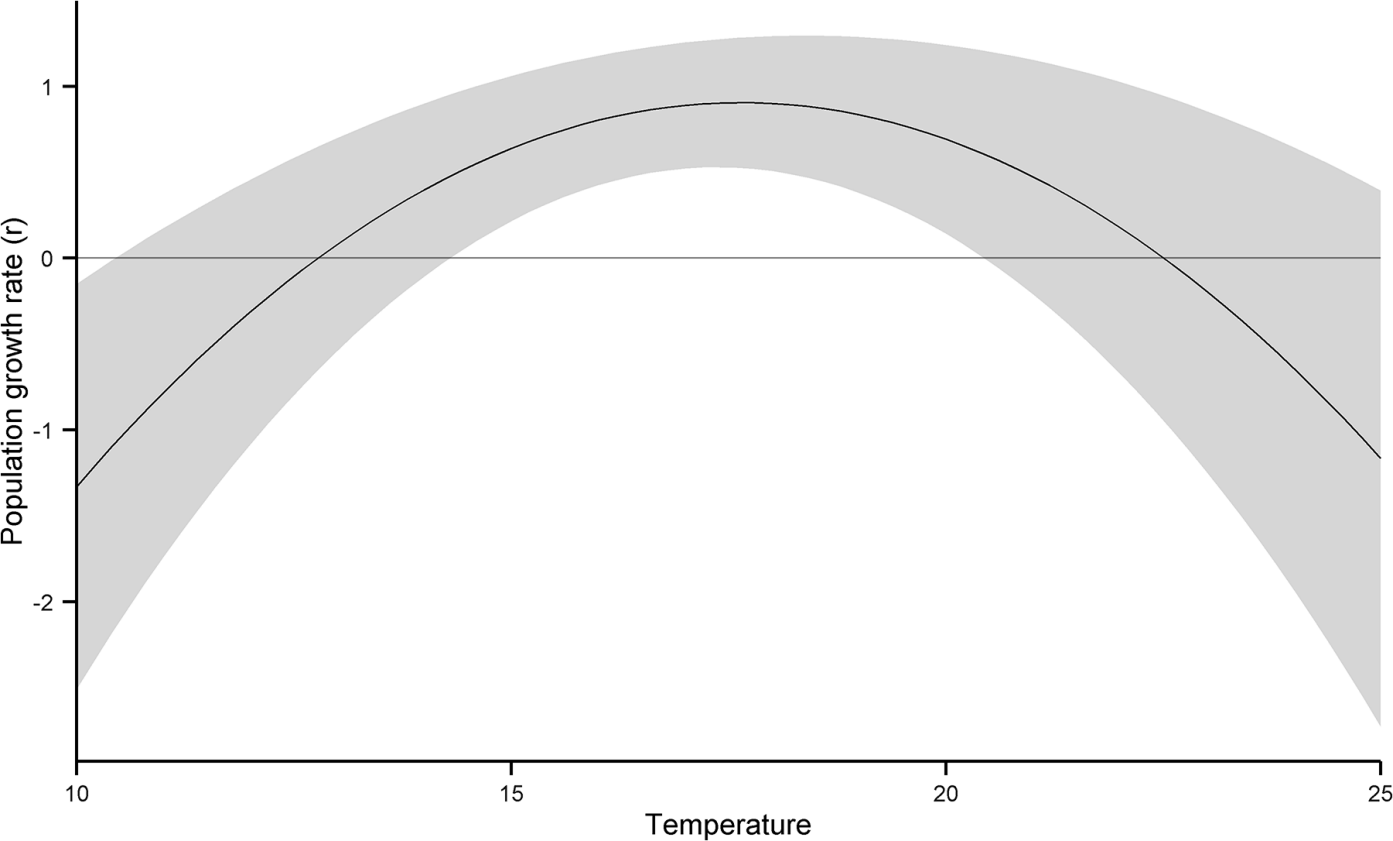
The fitted quadratic relationship between population growth rate and temperature (±s.e on each of the parameters α, β, γ). This underlying relationship was fitted according to Eq 12 and not directly to observations of annual population growth rate versus annual temperature. Note that the realised annual population growth rates will depend not just on annual temperature but also on intra-annual temperature variability and this graph shows the underlying relationship between annual population growth rate and annual temperature in the absence of this variability. Throughout the study period, there were 28 years in total over which annual population growth rate was quantified (i.e. N = 28).

**Fig 2:**
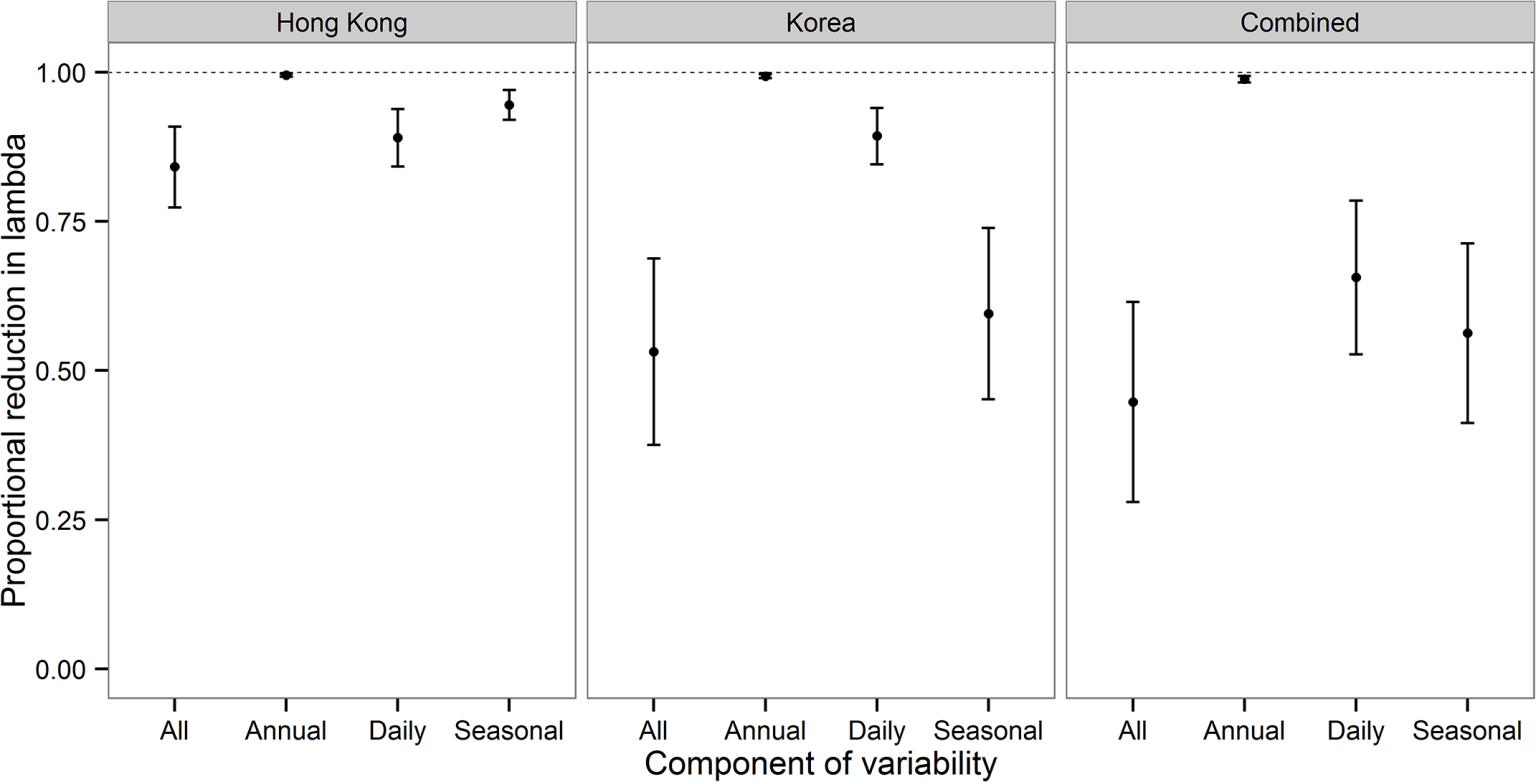
Proportional reduction in population multiplication rates caused by variability in temperature. In this figure, a value of 1 would indicate that variability had not reduced population multiplication rate below what could be achieved in a constant environment. A value of 0.75 would indicate that a particular component of variability had reduced the population multiplication rate by 25%. In this way, we estimated the effect of the various components of temperature variability–annual, daily, and seasonal–and further partitioned these effects into those caused by variability on the Hong Kong wintering grounds and those on the Korean breeding grounds. Together, all sources of temperature variability combined across Hong Kong and Korea can reduce population multiplication rate by about half, and much of this is due to the effect of seasonal variability in temperature on the Korean breeding grounds.

Our results show that the relationship between population growth rate and temperature is indeed nonlinear, with a significant negative quadratic component in the function (Table 1, Fig 1). The existence of this nonlinearity means that the variability in temperature can in principle influence the long-term average population growth rate, and our analyses then allowed us to quantify by how much. The combined effects of the annual, seasonal, and daily components of temperature variability in both Hong Kong and in Korea was large enough to drive annual population multiplication rate down to just 44.9% (± 13% SE) of what it could be if temperatures remained constant at their average values in Hong Kong and in Korea (Fig 2). The overall effect of temperature variability is thus considerable compared to previous studies and our analyses demonstrate here that wildlife population growth rates can be substantially reduced by environmental variation.

**Table 1:** Parameter estimates of population growth and temperature model.

Parameter	Estimate	Standard error	t-value	*P*-value
Intercept (α)	-11.0035	5.34819	-2.057	0.0512
Linear component (β)	1.34995	0.59374	2.274	0.0326
Quadratic component (γ)	-0.03826	0.01769	-2.163	0.0412
Detection *p*: ΔT_Summer_ (*b*)	-0.1689	0.15985	-1.057	0.3016
Detection *p*: ΔT_Winter_ (*c*)	0.07711	0.10087	0.764	0.4524

The relationship between population growth rate and temperature, as modelled using a quadratic function. The significant negative quadratic component indicates that the relationship between population growth rate and temperature is significantly nonlinear; increasing up to an optimum and then decreasing thereafter. The model also controls for possible effects of temperature on detection probability (Eq 11), but finds no evidence that these effects are actually significant in practice.

Our analyses further demonstrate that not all components of the temperature variance are equally important (Fig 2). Consistent with previous findings [17, 18], we show that the effects of inter-annual variability are actually very small, and a population exposed only to inter-annual variance would still be able to achieve an annual population multiplication rate which is 98.8% (±0.4% SE) of what it would achieve under constant average conditions. By contrast, a population exposed only to seasonal variation would be able to achieve an annual population multiplication rate which is only 56.4% (± 12% SE) of what could be achieved under constant conditions (Fig 2). Much of this effect was due to seasonal variation on the temperate Korean breeding grounds rather than on the tropical wintering grounds in Hong Kong. In tropical Hong Kong, the seasonal variation was smaller and less important than the daily variation. The effect of the daily variation in Korea was similar to the effect of daily variation in Hong Kong (Fig 2).

## Discussion

Our results show for the first time that short-term environmental variability can have a substantial effect on the long-term growth rates of a wildlife population. Although the theory has long indicated that this could be important in principle, it has rarely been tested, and here we provide the first empirical evidence that it is actually important in practice. We demonstrate that the nonlinearity in the relationship between *P*. *minor* population growth rate and temperature can indeed translate the prevailing levels of environmental variability into substantial effects on long-term average rates of population growth. Our analyses also demonstrate marked differences in the importance of different components of environmental variability, where annual variability had almost no effect while the seasonal variability in the temperate breeding grounds had a substantial impact.

These findings could have considerable implications for our understanding of population ecology more generally, highlighting a potentially much greater role for environmental variance in the population dynamics of wildlife. There is a large body of evidence that concave functions similar to those found here dominate the relationship between temperature and population growth in a wide range of species [4, 28, 29]. These underlying relationships have been particularly well-studied in ectotherms, and while the functions for ectotherms can be asymmetric becoming convex when temperatures fall far below the optimum, they show a concave function for most of the temperature range. In our study we have used a migratory endotherm which stays quite close to its optimum by migrating between temperate summers and tropical winters, and much like many tropical resident species that also live near their optimal temperatures, the relationship is consistently concave over this range of temperatures [4]. Although there will be some species whose population growth will follow convex relationships with temperature, particularly within the temperate zone, there are many which are concave like the black-faced spoonbill. If these concave relationships are similar to those we have found in our study system then they will also be capable of translating temperature variability into substantial negative impacts on long-term rates of population growth.

If temperature fluctuations are capable of reducing population growth rates by half, then environmental variability could be a major driver in the dynamics of populations. In this analysis, we isolated just one component of environmental variability, and the overall effect of environmental variability could be even greater given that wildlife populations are exposed to many other important environmental variables such as rainfall and ocean currents. As well as being important conceptually, these insights have important applications in conservation, highlighting the need to consider not just directional change in environmental conditions but also short-range stability and fluctuations. As the climate changes, much attention is being focused on the effects of change in average temperatures [30, 31], but changes in the variability could also be important. Climatic variability is more difficult to predict, but recent extreme weather events have been best explained by increased climate variability, and continued change is expected into the future [32]. Seasonal variability is expected to increase, while the daily range of temperature is expected to decrease, and any change in variability is expected to be spatially heterogeneous [33, 34]. Whatever the uncertainties, if current levels of temperature variability are already sufficient to halve the population multiplication rate of a high-profile endangered species, then attention needs to be paid not just to changing average temperatures but also to the population impact of changing environmental variability.

## Supporting Information

S1 File Data for construction of quadratic relationship between population multiplication rate (Nnx/N) and daily temperatures. See annotated analysis R-script (S3) for use of temperature variables in final model.
(CSV)

S2 File Data for modelling of climate scenarios which include either no, daily, seasonal, annual or all temperature variability. See annotated analysis R-script (S3) for use of temperature variables in final model.
(CSV)

S3 File Annotated R-script for construction of quadratic model and modelling the effect of daily, seasonal, annual and all temperature variability on population multiplication rate. (R)

## References

[1] Sibly RM, Hone J. Population growth rate and its determinants: an overview. Philosophical Transactions of the Royal Society of London B. 2002;357:1153–70. 10.1098/rstb.2002.1117 PMC1693026 12396508

[2] Schneider SH, Root TL. Wildlife responses to climate change: North American case studies Washington: Island Press; 2002.

[3] Thomas CD, Cameron A, Green RE, Bakkenes M, Beaumont LJ, Collingham YC, et al Extinction risk from climate change. Nature. 2004;427:145–8. 14712274 10.1038/nature02121

[4] Deutsch CA, Tewksbury JA, Huey RB, Sheldon KS, Ghalambor CK, Haak DC, et al Impacts of climate warming on terrestrial ectotherms across latitude. Proceedings of the National Academy of Sciences. 2008;105(18):6668–72. 10.1073/pnas.0709472105 PMC2373333 18458348

[5] Lundberg P, Ranta E, Ripa J, Kaitala V. Population variability in space and time. Trends in Ecology and Evolution. 2000;15(11):460–4. 11050349 10.1016/s0169-5347(00)01981-9

[6] Lande R. Risks of population extinction from demographic and environmental stochasticity and random catastrophes. The American Naturalist. 1993;142(6):911–27. 10.1086/285580 29519140

[7] Preston KL, Redak RA, Allen MF, Rotenberry JT. Changing distribution patterns of an endangered butterfly: Linking local extinction patterns and variable habitat relationships. Biological Conservation. 2012;152:280–90.

[8] Boyce MS, Haridas CV, Lee CT, NCEAS Stochastic Demography Working Group. Demography in an increasingly variable world. Trends in Ecology and Evolution. 2006;21(3):141–8. 16701490 10.1016/j.tree.2005.11.018

[9] Lewontin RC, Cohen D. On population growth in a randomly varying environment. Proceedings of the National Academy of Sciences. 1969;62(4):1056–60. 10.1073/pnas.62.4.1056 PMC223613 5256406

[10] Jensen JLWV. Sur les fonctions convexes et les inégalités entre les valeurs moyennes. Acta Mathematica. 1906;30(1):175–93.

[11] Ruel JJ, Ayres MP. Jensen's inequality predicts effects of environmental variation. Trends in Ecology and Evolution. 1999;14(9):361–6. 10441312 10.1016/s0169-5347(99)01664-x

[12] Doak DF, Morris WF, Pfister CA, Kendall BE, Bruna EM. Correctly estimating how environmental stochasticity influences fitness and population growth. American Naturalist. 2005;166(1):E14–E21. 15937784 10.1086/430642

[13] Drake JM. Population effects of increased climate variation. Proceedings of the Royal Society of London B. 2005;272(1574):1823–7. 10.1098/rspb.2005.3148 PMC1559868 16096095

[14] Boyce MS. Population-growth with stochastic fluctuations in the life table. Theoretical Population Biology. 1977;12(3):366–73. 601721 10.1016/0040-5809(77)90050-8

[15] Cohen D. A general model of optimal reproduction in a randomly varying environment. Journal of Ecology. 1968;56:219–28.

[16] Huey RB, Berrigan D. Temperature, demography, and ectotherm fitness. American Naturalist. 2001;158:204–10. 10.1086/321314 18707349

[17] van de Pol M, Vindenes Y, Saether B, Engen S, Ens BJ, Oosterbeek K, et al Effects of climate change and variability on population dynamics in a long-lived shorebird. Ecology. 2010;91:1192–204. 20462133 10.1890/09-0410.1

[18] Morris WF, Pfister CA, Tujiapurkar S, Haridas CV, Boggs CL, Boyce MS, et al Longevity can buffer plant and animal populations against changing climatic variability. Ecology. 2008;89(1):19–25. 18376542 10.1890/07-0774.1

[19] Carey GJ. Waterbird count handbook: A guide for participants in the waterbird counts in Hong Kong 2nd ed Hong Kong: Hong Kong Bird Watching Society; 2002.

[20] Kalnay E, Kanamitsu M, Kistler R, Collins W, Deavan D, Gandin L, et al The NCEP/NCAR 40-year reanalysis project. Bulletin of the American Meteorological Society. 1996;77(3):437–71.

[21] Ueta M, Melville DS, Wang Y, Ozaki K, Kanai Y, Leader PJ, et al Discovery of the breeding sites and migration routes of Black-faced Spoonbill Platalea minor. Ibis. 2002;144(2):340–3.

[22] McKechnie AE, Wolf BO. Climate change increases the likelihood of catastrophic avian mortality events during extreme heat waves. Biology Letters. 2009; 10.1098/rsbl.2009.0702 PMC2865035 19793742

[23] Cawthorne RA, Marchant JH. The effects of the 1978/79 winter on British bird populations. Bird Study. 1980;27(3):163–72.

[24] Robb GN, McDonald RA, Chamberlain DE, Reynolds SJ, Harrison TJE, Bearhop S. Winter feeding of birds increases productivity in subsequent breeding season. Biology Letters. 2008; 10.1098/rsbl.2007.0622 PMC2429937 18252663

[25] Ueng Y-T, Perng J-J, Wang J-P, Weng J-H, Hou P-CL. Diet of the black-faced spoonbill wintering at Chiku Wetland in Southwestern Taiwan. Waterbirds. 2006;29(2):185–90.

[26] R Core Team. R: A language and environment for statistical computing. 2013.

[27] Powell LA. Approximating variance of demographic parameters using the delta method: a reference for avian biologists. The Condor. 2007;109(4):949–54.

[28] Frazier MR, Huey RB, Berrigan D. Thermodynamic constraints on the evolution of insect growth rates: "warmer is better". American Naturalist. 2006;168:512–20. 17004222 10.1086/506977

[29] Amarasekare P, Savage V. A framework for elucidating the temperature dependence of fitness. American Naturalist. 2012;179(2):178–91. 10.1086/663677 22218308

[30] Walther G-R, Post E, Convey P, Menzel A, Parmesan C, Beebee TJC, et al Ecological responses to recent climate change. Nature. 2002;416:389–95. 11919621 10.1038/416389a

[31] IPCC. Climate change 2014: Impacts, Adaptation and Vulnerability Contribution of Working Group II to the Fifth Assessment Report of the Intergovernmental Panel on Climate Change: Cambridge University Press, Cambridge, United Kingdom; 2014.

[32] Schär C, Vidale PL, Lüthi D, Frei C, Häberli C, Liniger MA, et al The role of increasing temperature variability in European summer heatwaves. Nature. 2004;427:332–6. 14716318 10.1038/nature02300

[33] Giorgi F, Bi X, Pal J. Mean, interannual variability and trends in regional climate change experiments over Europe. II: climate change scenarios (2071–2100). Climate Dynamics. 2004;23(7–8):839–58.

[34] Karl TR, Jones PD, Knight RW, Kukla G, Plummer N, Razuvayev V, et al Asymmetric trends of daily maximum and minimum temperature. Bulletin of the American Meteorological Society. 1993;74(6):1007–23.

